# Sjögren’s Disease With Primary Biliary Cholangitis Fulfilling the 2010 ACR/EULAR Classification Criteria for Rheumatoid Arthritis: A Case Report and Review of an Under-Recognized Overlap

**DOI:** 10.7759/cureus.117335

**Published:** 2026-10-01

**Authors:** Carolina Ejnisman, Briane Pasini, Henrique S Shiroma, André B Hirayama, Sandra G Pasoto, André S Franco

**Affiliations:** 1 Rheumatology Division, Hospital das Clínicas da Faculdade de Medicina da Universidade de São Paulo, São Paulo, BRA; 2 Gastroenterology and Hepatology, Universidade Estadual de Mato Grosso do Sul, Campo Grande, BRA; 3 Pathology, Hospital das Clínicas da Faculdade de Medicina da Universidade de São Paulo, São Paulo, BRA

**Keywords:** classification criteria, primary biliary cirrhosis (pbc), rheumatoid arthritis, rheumatoid factor (rf), sjogren's disease

## Abstract

Sjögren’s disease (SjD) and primary biliary cholangitis (PBC) are chronic autoimmune epithelitides that target exocrine glands and small intrahepatic bile ducts, respectively. Their coexistence is well described; less appreciated is a specific and avoidable failure mode. Because rheumatoid factor (RF) positivity, symmetric small-joint involvement and elevated acute-phase reactants are all common in SjD, patients with SjD-associated polyarthritis may reach the six-point threshold of the 2010 American College of Rheumatology/European League Against Rheumatism (ACR/EULAR) classification criteria for rheumatoid arthritis (RA); those criteria remain valid only if their mandatory exclusion clause is applied.

We report a 49-year-old woman who carried a three-year diagnosis of RF-positive, anti-citrullinated peptide antibody (ACPA)-negative RA and had been treated with methotrexate and tofacitinib without ever developing radiographic erosions. Cholestatic liver injury during a course of amoxicillin-clavulanate led to the detection of anti-mitochondrial M2 antibodies and to a liver biopsy showing florid duct lesions with periductal granulomas, establishing stage 1 PBC. Directed questioning subsequently revealed a three-year history of previously unreported sicca symptoms. Anti-Sjögren’s-syndrome-related antigen A autoantibodies (anti-Ro/SSA) were positive, the Schirmer test measured 1 mm bilaterally, and minor salivary gland biopsy demonstrated focal lymphocytic sialadenitis with a focus score of 2. These findings yielded a total of 7/9 points according to the 2016 ACR/EULAR classification criteria for SjD, with relevant exclusion criteria assessed and found to be absent.

Immunosuppression was withdrawn, followed by sustained biochemical and articular improvement over 24 months after initiation of ursodeoxycholic acid and hydroxychloroquine. We review the epidemiology, shared immunopathogenesis, and therapeutic implications of the SjD-PBC overlap, and propose that, in an appropriate clinical context, a structured inquiry into sicca symptoms and consideration of anti-Ro/SSA testing may be useful in patients with ACPA-negative, RF-positive, non-erosive polyarthritis before escalation to biologic or targeted synthetic therapy.

## Introduction

Sjögren’s disease (SjD) and primary biliary cholangitis (PBC) are chronic autoimmune disorders in which the primary target is epithelium: the ductal and acinar epithelium of the salivary and lacrimal glands in SjD, and the biliary epithelium of the small intrahepatic bile ducts in PBC. The unifying concept of autoimmune epithelitis rests on their shared histological architecture, their comparable epidemiology, onset in the fifth decade, marked female predominance, and an accumulating body of genetic and immunological data [1-3]. Throughout this report, we use the term Sjögren’s disease and the descriptor associated rather than secondary, in accordance with the 2023 International Rome consensus on nomenclature [4].

Although the association between SjD and PBC is well recognized, this report addresses a more practical clinical issue: the potential misclassification of SjD-associated inflammatory arthritis as rheumatoid arthritis (RA). Articular involvement is the most common extraglandular manifestation of SjD and may precede sicca symptoms. Because patients frequently present with symmetric polyarthritis, rheumatoid factor positivity, and elevated inflammatory markers, they may fulfil the 2010 American College of Rheumatology/European League Against Rheumatism (ACR/EULAR) classification criteria for RA [5-7].

Importantly, these criteria require not only clinical synovitis but also the exclusion of a more appropriate alternative diagnosis before classification [7]. Thus, the challenge lies not in the criteria themselves but in their application, particularly when sicca symptoms are overlooked and the exclusion step is insufficiently considered.

We report a patient in whom this sequence unfolded over three years and was interrupted only when an unrelated drug exposure precipitated cholestatic liver injury, leading to the diagnosis of PBC and thence to SjD. We pair the case with a review of the SjD-PBC overlap weighted toward the questions the case raises: how to separate SjD-associated polyarthritis from a genuine RA-SjD overlap, how much of the shared-pathogenesis literature actually supports a specific link, and what the therapeutic consequences of misclassification are. The case is reported in line with the CARE reporting guideline [8].

## Case presentation

A 49-year-old woman of mixed ancestry, with no relevant family history of autoimmune disease and no history of alcohol use, was referred to our tertiary rheumatology center with a three-year diagnosis of rheumatoid arthritis (RA) established at another institution.

According to the available medical records, her initial presentation consisted of inflammatory polyarthralgia involving small and large joints, associated with morning stiffness lasting more than one hour and symptom duration of more than six weeks. Rheumatoid factor (RF) was strongly positive (128 mg/L), anti-citrullinated peptide antibodies (ACPA) were negative, the erythrocyte sedimentation rate was 96 mm/h, and C-reactive protein was 4.54 mg/L (Table 1). The diagnosis of RA was made based on these findings.

**Table 1:** Laboratory findings at initial rheumatologic assessment, during the cholestatic episode, and at rheumatologic reassessment.

Parameters	Timing	Patient values	Reference range
Rheumatoid factor (RF)	Initial rheumatologic assessment	128 mg/L	<8 mg/L
Anti-cyclic citrullinated peptide antibodies (ACPA)	Initial rheumatologic assessment	Negative	Negative
Erythrocyte sedimentation rate (ESR)	Initial rheumatologic assessment	96 mm/h	<10 mm/h
C-reactive protein (CRP)	Initial rheumatologic assessment	4.54 mg/L	<5 mg/L
Aspartate aminotransferase (AST)	Cholestatic episode	115 U/L	10–37 U/L
Alanine aminotransferase (ALT)	Cholestatic episode	148 U/L	10–37 U/L
Alkaline phosphatase (ALP)	Cholestatic episode	341 U/L	24–118 U/L
Gamma-glutamyl transferase (GGT)	Cholestatic episode	431 U/L	5–39 U/L
Total bilirubin	Cholestatic episode	12.58 mg/dL	<1.0 mg/dL
Viral hepatitis serology	Cholestatic episode	Negative	Negative
Anti-mitochondrial M2 antibodies (AMA-M2)	Cholestatic episode	199 IU/mL	<20 IU/mL
Anti-Ro/SSA antibodies	Rheumatologic reassessment	Positive	Negative

However, the original records did not include a detailed joint examination with swollen and tender joint counts, and no ultrasound or magnetic resonance imaging documenting synovitis was available. Conventional radiographs of the hands, wrists, and feet showed no erosions or periarticular osteopenia. Sicca symptoms were not investigated. Methotrexate (15 mg/week) was initiated but discontinued because of gastrointestinal intolerance, and tofacitinib (5 mg twice daily) was subsequently prescribed. Although symptoms fluctuated, complete remission was never achieved.

Three years later, during a 10-day course of amoxicillin-clavulanate (875 mg twice daily) for sinusitis, the patient developed intense pruritus and jaundice. Laboratory tests revealed a predominantly cholestatic liver injury pattern (R factor=1.4; Table 1). Viral hepatitis serologies were negative, and abdominal ultrasonography excluded biliary obstruction. Despite discontinuation of both amoxicillin-clavulanate and tofacitinib, liver biochemistry remained abnormal. Anti-mitochondrial M2 antibodies were strongly positive (199 IU/mL), and liver biopsy demonstrated florid duct lesions, periductal granulomas, and mild portal fibrosis, establishing the diagnosis of stage 1 primary biliary cholangitis (PBC) (Figures 1-2).

**Figure 1:**
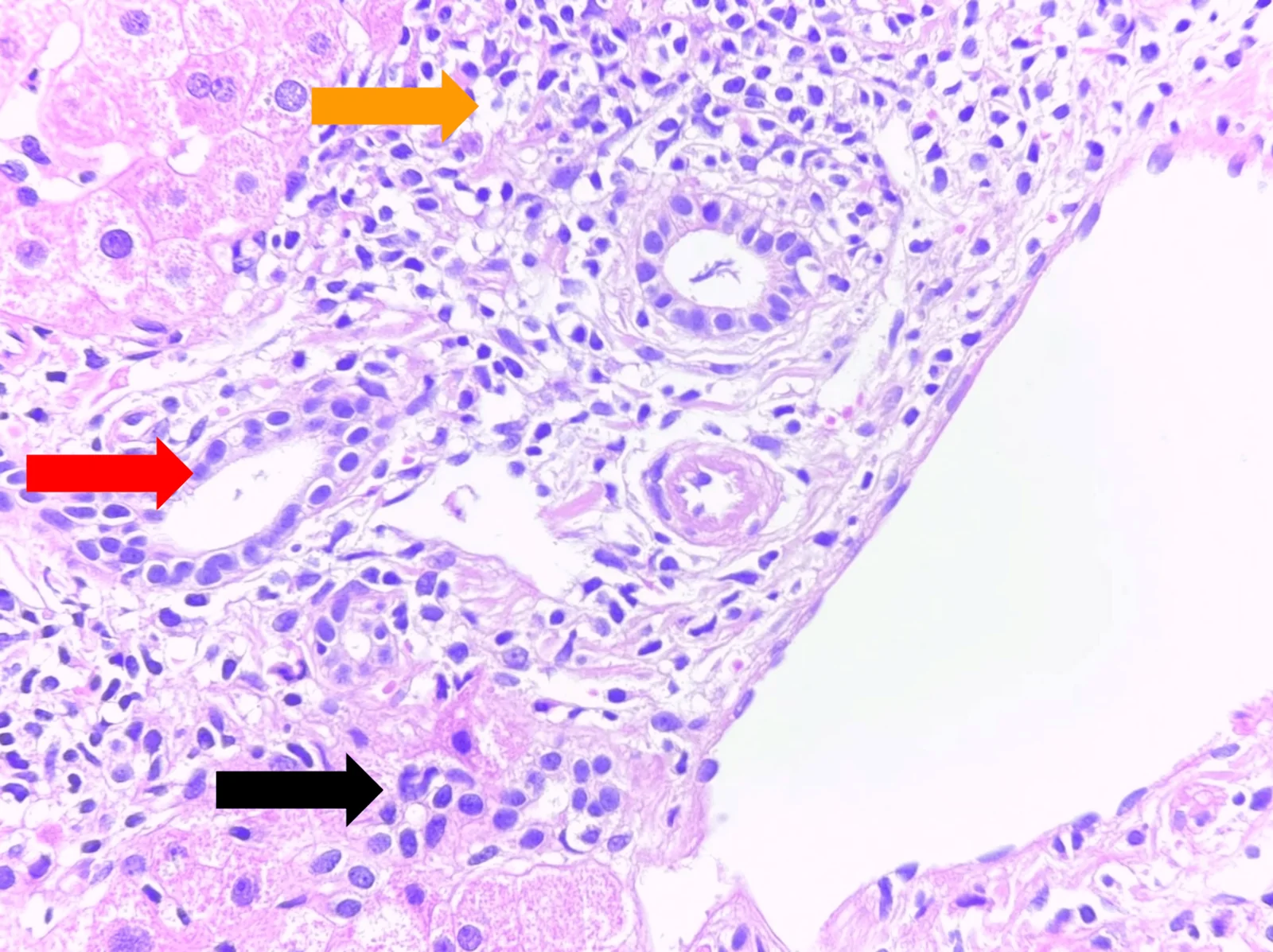
Liver biopsy. Photomicrography showing portal lymphohistiocytic inflammation (orange arrow) with disruption of biliary epithelium (red arrow) and ductular reaction (black arrow); haematoxylin and eosin, 400x.

**Figure 2:**
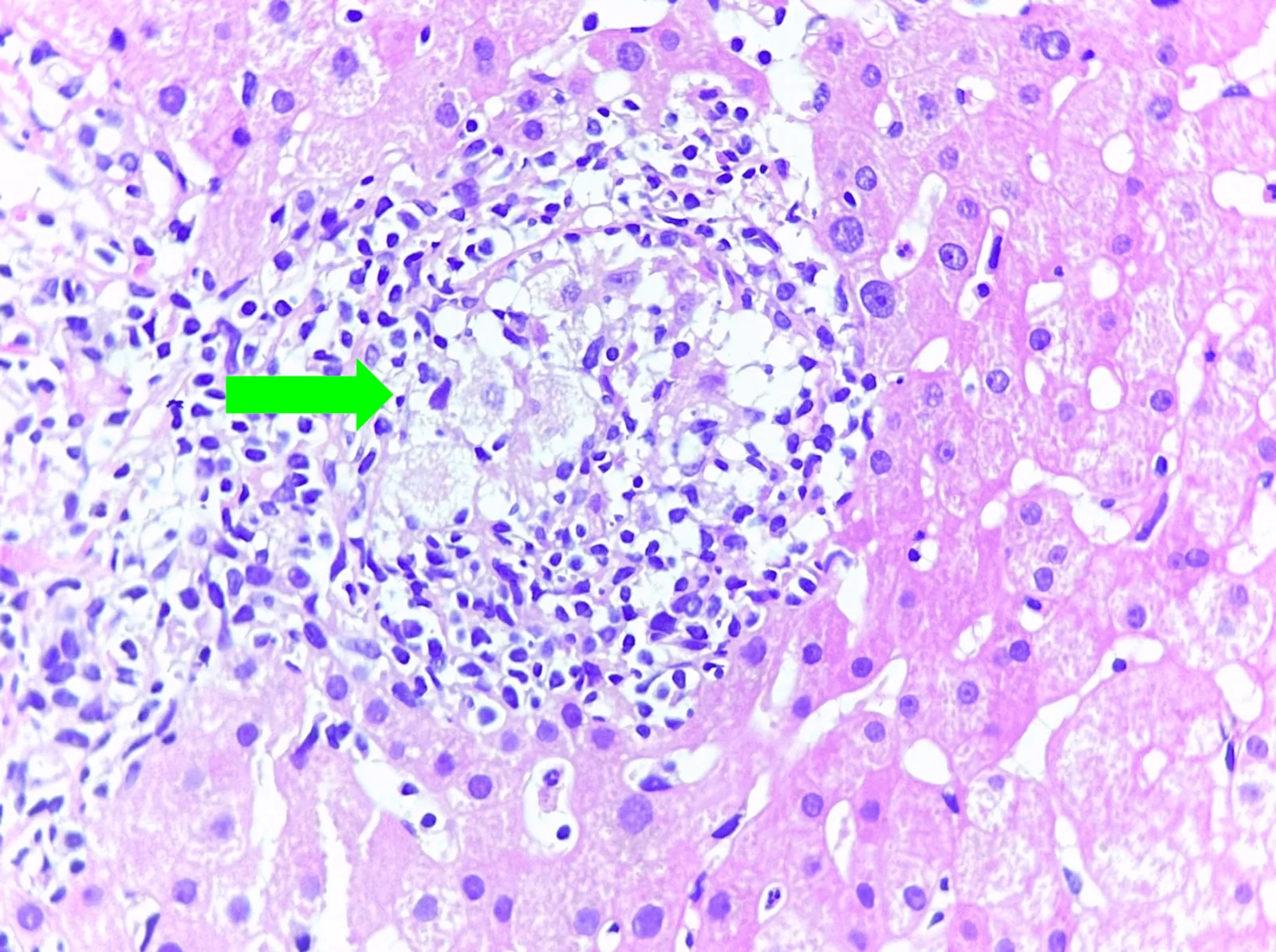
Liver biopsy. Periductal granulomatous reaction (green arrow); haematoxylin and eosin, 400x.

The diagnosis of PBC prompted a comprehensive rheumatologic reassessment. Directed questioning revealed that xerostomia and xerophthalmia had begun simultaneously with the onset of joint symptoms three years earlier. Although dry eye had been diagnosed by an ophthalmologist at that time, this information had not been incorporated into the rheumatologic evaluation. Anti-Ro/SSA antibodies were positive, Schirmer's test measured 1 mm bilaterally, unstimulated salivary flow was 0.2 mL/min, and minor salivary gland biopsy showed focal lymphocytic sialadenitis with a focus score of 2, fulfilling the 2016 ACR/EULAR classification criteria for Sjögren's disease [9].

At reassessment in our service, no joint fulfilled the definition of clinical synovitis. Review of the available records suggested that the initial diagnosis of RA had relied mainly on the distribution of joint symptoms, high-titer RF, elevated erythrocyte sedimentation rate, and symptom duration. However, objective documentation of synovitis at disease onset was unavailable, and no imaging studies confirmed inflammatory arthritis. Furthermore, serial radiographs obtained during follow-up remained free of erosive changes, arguing against previous erosive RA. The diagnosis was therefore revised to Sjögren's disease-associated inflammatory polyarthralgia with coexisting PBC.

Ursodeoxycholic acid was started at 900 mg/day (12.7 mg/kg/day) and hydroxychloroquine at 5 mg/kg/day. Preservative-free ocular lubricants and topical cyclosporine 0.05% four times daily were prescribed for keratoconjunctivitis sicca, with pilocarpine 5 mg three times daily for xerostomia and sodium fluoride 1.1% gel for caries prophylaxis after a carious lesion was identified. Liver biochemistry improved rapidly, with alkaline phosphatase falling to 182 U/L (reference range 24-118 U/L), gamma-glutamyl transferase to 33 U/L (reference range 5-39 U/L), and total bilirubin to 1.78 mg/dL (reference range <1.0 mg/dL). Inflammatory arthralgia also resolved.

At the most recent review, 24 months after reclassification, musculoskeletal examination showed no synovitis in any joint; liver enzymes remained normal; mild pruritus persisted; unstimulated salivary flow was unchanged at 0.2 mL/min. Disease activity, assessed using the EULAR Sjögren's Syndrome Disease Activity Index (ESSDAI) [10], was zero. No systemic immunosuppression beyond hydroxychloroquine has been required, and no erosive disease has developed on serial radiographs.

## Discussion

Relevant literature for the review component of this report was identified through a structured search of MEDLINE/PubMed using the terms "Sjögren" AND "primary biliary cholangitis." The search retrieved 450 articles published between 1968 and 2025. Original studies, systematic reviews, and meta-analyses addressing the epidemiological, clinical, and immunological overlap between Sjögren’s disease (SjD) and primary biliary cholangitis (PBC) were considered eligible. Studies with unrelated primary objectives and those not published in English were excluded. After screening titles and abstracts, 19 articles were selected for full-text review. Two articles were subsequently excluded because they were not published in English. The remaining 17 articles were included in the narrative review. To ensure comprehensive coverage of the topic, a manual search of the reference lists of these articles was performed, identifying six additional relevant studies. In total, 23 articles addressing the SjD-PBC overlap were included in the review. Additional references related to disease classification criteria, treatment recommendations, and background information were cited separately, as appropriate.

**Figure 3:**
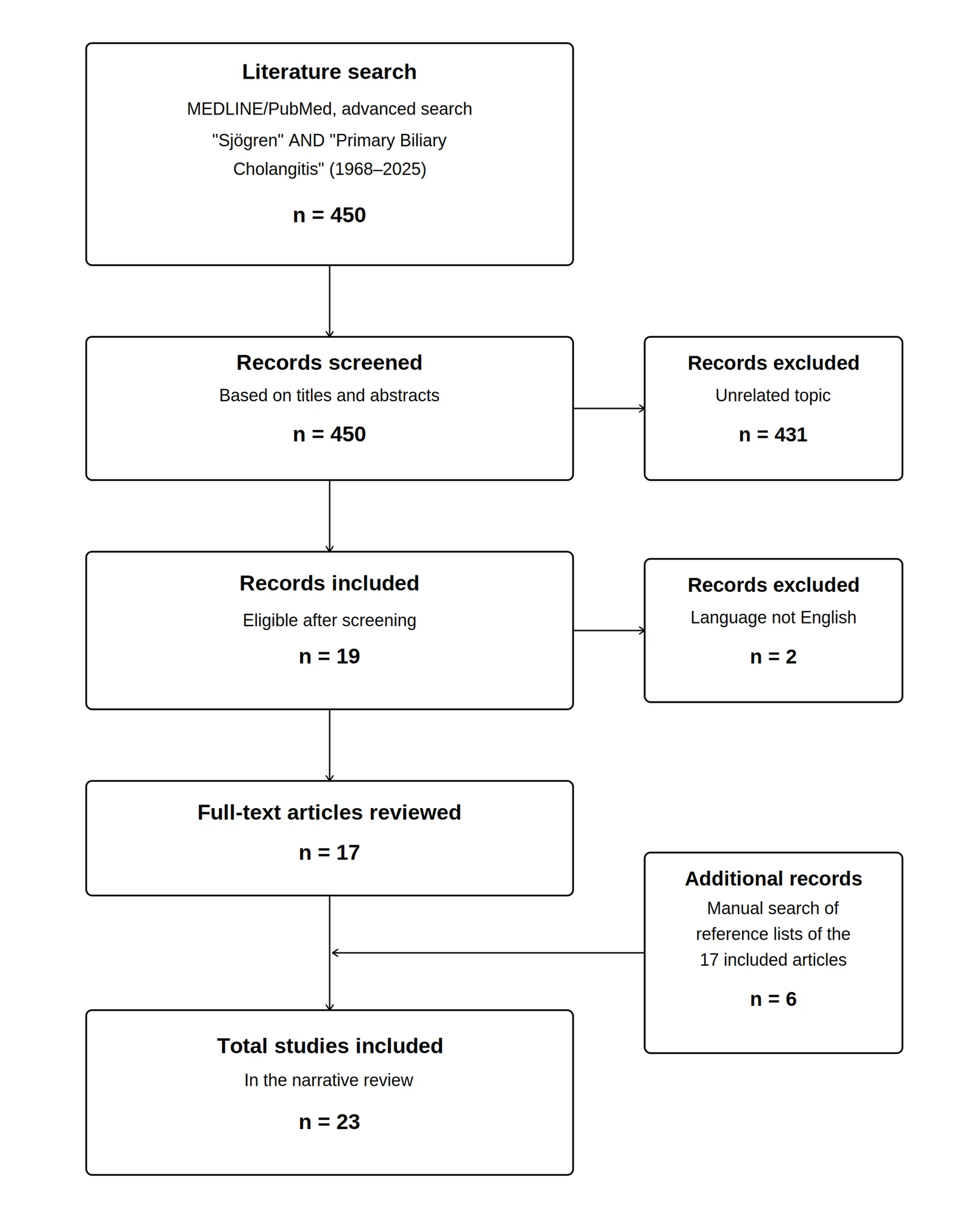
Flow diagram of the literature search and study selection MEDLINE/PubMed advanced search combining the terms “Sjögren” and “Primary Biliary Cholangitis” (1968-2025) yielded 450 records, screened by title and abstract; two were excluded for language, 19 records were included and 17 full-text articles were reviewed. Six additional records were identified through manual search of reference lists, for a total of 23 studies included in the narrative review.

2010 criteria classified this patient as having RA

The 2010 ACR/EULAR criteria were derived in cohorts of patients presenting with undifferentiated early inflammatory synovitis, and were explicitly optimized to identify those at risk of persistent or erosive disease, that is, to increase sensitivity in early disease, where the 1987 criteria performed poorly [7,11]. Two design decisions follow from that objective and matter here: the criteria trade specificity for sensitivity by design, and they delegate specificity to the exclusion clause rather than to the score.

What separates the two diagnoses is therefore not in the score at all. It is in the gate: definite clinical synovitis in at least one joint, and no better alternative explanation [7]. In this case, neither prerequisite had been documented. The absence of erosions on serial radiographs over three years, negative ACPA, an incomplete response to two disease-modifying agents, and, importantly, a previously unelicited three-year history of sicca symptoms collectively supported an alternative diagnosis that better explained the synovitis and should therefore be excluded. The patient’s failure to meet the 1987 criteria, which require objective arthritis and allow for radiographic and nodular findings, is consistent with this interpretation.

We draw a practical conclusion that we have not seen stated explicitly in the overlap literature: in the ACPA-negative, RF-positive patient, the 2010 score should be regarded as necessary but not sufficient, and a structured sicca inquiry plus anti-Ro/SSA testing should be treated as part of the exclusion step rather than as an optional refinement. This is a low-cost intervention with a defined decision point - before escalation beyond conventional synthetic disease-modifying antirheumatic drugs (DMARDs).

Distinguishing SjD-associated polyarthritis from a true RA-SjD overlap

The distinction matters because the two entities differ in prognosis and in treatment, and recent work has begun to give it empirical content. Aradi and colleagues stratified 164 patients with SjD into those with polyarthritis as an extraglandular manifestation, those with coexisting RA, and those without inflammatory joint pain, and found that RF isotype profiles carried additive discriminative value: the combination of high total IgG with high IgA RF characterized SjD, whereas normal IgG with high IgM RF was significantly more frequent in the RA-associated group [12]. Gan and colleagues addressed the same question with machine learning in 241 patients and reported that a random forest model separated RA-SjD overlap from SjD with polyarthritis with an area under the curve of 0.854, the dominant discriminators being anti-CCP level, RF level, erosive joint count, anti-Ro60 antibodies and C-reactive protein [13].

Two observations follow. First, both studies converge on erosion and ACPA as the features that carry greater discriminatory value-exactly the two features that were absent in our patient. Second, and more soberly, both are single-center retrospective studies without external validation, and the machine learning model in particular is at evident risk of overfitting at this sample size. They support a clinical heuristic; they do not yet support an algorithm. In the absence of validated tools, serial radiography, ACPA, and a documented sicca history remain the practical discriminators.

A caveat applies to the weight placed on non-erosive as a discriminator, and it cuts in both directions. Radiographic erosions are reported in approximately 1.5% of patients with SjD, while ultrasonography detects erosions in 27-35%. In a previous study of 97 patients with SjD in whom RA had been formally excluded, one of the authors used high-resolution peripheral quantitative computed tomography and identified erosions in 56.7% of patients, with a mean erosion volume of 5.71 mm³ [14]. Two findings from that study qualify how such lesions should be read. Erosion prevalence among age- and body mass index-matched healthy women was essentially identical (58.3%), and only erosion volume separated the groups (5.71 versus 0.38 mm³, p< 0.001), so the mere presence of a subcortical break on sensitive imaging is not disease-specific. Moreover, erosion volume tracked age, postmenopausal status and cortical bone parameters rather than ESSDAI, damage or functional scores, indicating that these lesions do not carry the prognostic meaning that erosions carry in RA. Non-erosive should therefore be read as a statement about plain radiography, the modality on which the 1987 criteria and routine practice rely, rather than as a claim that the SjD joint is structurally intact.

Epidemiology: what the prevalence figures actually mean

SjD has an estimated incidence of 3.5-3.9 per 100,000 person-years and a female-to-male ratio near 9:1 [15]; PBC is rarer, at approximately 1.76 per 100,000, with a female predominance of 4-6:1 [16,17]. The association between them is the strongest of the autoimmune hepatobiliary overlaps, and is conventionally quoted as affecting 3-9% of patients with SjD [18,19].

That range deserves scrutiny rather than repetition, because recent cohorts diverge sharply. Park and colleagues reviewed 1119 patients with SjD in Korea and found autoimmune hepatic involvement in 51 (4.6%), of whom PBC accounted for 27.5% and an autoimmune hepatitis-PBC overlap for a further 7.8%, implying a PBC prevalence of roughly 1.6% [20]. Liang and colleagues, in a 10-year Chinese cohort of 568 patients, identified SjD-associated autoimmune liver disease in 117 (20.6%) [21]. An order of magnitude separates these estimates.

The explanation is almost certainly definitional and methodological rather than biological. Studies differ in the classification criteria applied, in whether autoimmune liver disease is defined serologically or histologically, in whether anti-mitochondrial antibodies are screened for systematically or tested only when liver enzymes are abnormal, and in referral patterns, since tertiary hepatology-affiliated cohorts will enrich for hepatic involvement. Our own case illustrates the ascertainment problem directly: had the antibiotic exposure not occurred, this patient’s PBC would in all likelihood have remained subclinical and unrecorded. Consistent with this, among 368 consecutive patients screened at our own SjD outpatient clinic, PBC was documented in only two (0.5%) [14], a figure derived from case-note review rather than from systematic anti-mitochondrial antibody testing, and therefore a lower bound set by ascertainment rather than a prevalence estimate. Until a study applies uniform criteria with systematic screening in an unselected SjD population, the true prevalence should be regarded as unknown and the 3-9% figure treated as a convention rather than an estimate.

Two serological markers do appear robust across studies. Gao and colleagues identified elevated liver enzymes, raised immunoglobulins (IgM, IgG2, IgG3) and high-titer antinuclear antibodies as laboratory risk factors for coexistent PBC in SjD [22]. In the reciprocal direction, Dahl and colleagues found anti-Ro52/TRIM21 antibodies in 16.9% of 236 Danish patients with PBC and identified 14 patients within that subgroup who met criteria for SjD but had not been diagnosed [23], a finding that argues for rheumatological referral of anti-Ro52-positive patients with PBC, though it awaits replication outside a single national cohort.

Shared immunopathogenesis: the evidence and its limits

The mechanistic literature linking SjD and PBC is substantial, and it is worth separating what it establishes from what it merely suggests. Genome-wide association studies have identified susceptibility loci shared by the two diseases, including HLA class II variants and non-HLA genes in the IL-12/IL-23 axis (IL12A, IL12RB2, STAT4) and in B-cell activation (TNFSF4, BLK) [24-29]. The critical caveat is that most of these are generic autoimmunity loci, shared across systemic lupus erythematosus, systemic sclerosis and RA as well: they establish a common susceptibility architecture for humoral autoimmunity, not a specific SjD-PBC axis. A two-sample Mendelian randomization study reported that genetic liability to PBC increases the risk of SjD by approximately 18% (OR = 1.18, 95% CI 1.12-1.24, p< 0.001) [30], but the analysis was restricted to European-ancestry populations, and directional and pleiotropy assumptions are difficult to satisfy given the extensive locus sharing just described.

Transcriptomic analysis of publicly available datasets identified CX3CR1, ELF1, CUL1, PARP3, DDX60, SP140L and MUC1 as crosstalk genes between the two diseases [31]. Several of these, DDX60 and CX3CR1 in particular, are interferon-inducible, so their co-expression may reflect a shared type I interferon signature common to many systemic autoimmune diseases rather than a mechanism unique to this pair, and the analysis used bulk peripheral blood rather than target tissue. Reduced alpha diversity and depletion of Bacteroides have been described in both diseases, with disease-specific enrichment of Actinobacteria in SjD and Proteobacteria in PBC [32]; that study is cross-sectional and cannot separate dysbiosis as cause from dysbiosis as consequence of disease or of ursodeoxycholic acid and immunosuppressive exposure.

The mechanistic account with the strongest tissue-level support concerns autoantigen exposure during epithelial apoptosis. In PBC, the E2 subunit of the pyruvate dehydrogenase complex remains immunologically intact within apoptotic blebs of biliary epithelium and becomes accessible to anti-mitochondrial antibodies [33]; in SjD, Ro and La antigens are similarly translocated to the apoptotic cell surface [34]. Both biliary and salivary epithelial cells display premature senescence with telomere shortening and p16 expression, together with oxidative stress, generating a pro-inflammatory secretory phenotype [35,36]. Downstream, Th17/Treg imbalance correlates with hepatic dysfunction in PBC and glandular inflammation in SjD [37], with impaired Treg function linked to FoxP3 demethylation [38,39], and B-cell activating factor (BAFF) overexpression is a hallmark of both, correlating with autoantibody titers and disease activity [40,41]. Environmental factors have been proposed as potential triggers for both conditions, including Epstein-Barr virus (EBV), which is tropic for glandular epithelium [42], and, for PBC specifically, molecular mimicry between PDC-E2 and antigens of Escherichia coli and Novosphingobium aromaticivorans [43,44], with xenobiotic modification of epithelial proteins as a further candidate mechanism [45].

Taken together, this literature supports a common effector program acting on epithelium, apoptotic autoantigen exposure, senescence, T-cell imbalance and B-cell hyperactivity with considerably weaker evidence for a disease-pair-specific upstream trigger. The distinction is not academic: it predicts that therapies targeting the shared effector program should benefit both diseases, whereas the search for a unique SjD-PBC mechanism may not be productive.

Therapeutic implications of misclassification

The consequences of a mistaken RA label in this setting are concrete rather than nosological. For PBC, ursodeoxycholic acid at 13-15 mg/kg/day remains first-line, improving biochemical markers and delaying histological progression [46]; coexisting SjD does not impair the hepatic response [47]. For SjD, treatment of sicca symptoms is primarily symptomatic, while systemic therapy is generally reserved for patients with moderate-to-high systemic activity. Hydroxychloroquine is commonly used in clinical practice for inflammatory arthralgia and fatigue, although evidence of efficacy for these SjD-related symptoms from randomized controlled trials remains limited [48]. Our patient experienced sustained biochemical improvement following treatment for PBC and complete resolution of articular symptoms after the introduction of hydroxychloroquine. However, treatment response should not be considered diagnostic; these clinical responses were consistent with the subsequent diagnoses.

Conversely, she received three years of therapy calibrated to a disease she did not have. Methotrexate was poorly tolerated, and tofacitinib was associated with recurrent infection; the eventual hepatic event occurred on a background of undiagnosed PBC, and although amoxicillin-clavulanate is the most probable proximate trigger, the relative contributions of the antibiotic, the JAK inhibitor and unmasking of pre-existing cholestatic disease cannot be disentangled retrospectively. The relevant point is that the risk was taken unnecessarily.

A further consideration strengthens the case for looking before escalating. Horai and colleagues reviewed the evidence that RA with coexisting SjD or anti-Ro/SSA positivity behaves as a distinct phenotype, with reduced responsiveness to several DMARD classes and an elevated risk of lymphoproliferative disorder, particularly with methotrexate [49]. Identifying SjD before starting a biologic or targeted synthetic disease-modifying antirheumatic drug (DMARD) therefore alters drug selection and risk stratification even when a genuine RA overlap is present. Looking forward, the shared effector pathways identified above are already therapeutic targets: ianalumab, a BAFF-receptor-directed antibody, sustained efficacy through 52 weeks in a phase 2b dose-ranging study in SjD [50], and agents acting on the IL-12/23 axis or on B-cell survival are plausible cross-over interventions [34]. None has been evaluated in the SjD-PBC overlap specifically.

Unresolved questions

Whether all patients with SjD should undergo anti-mitochondrial antibody screening at diagnosis, and whether testing should be repeated over time, remains an unresolved question. These antibodies may precede biochemical PBC by years, and our case shows that alternative testing only when liver enzymes are abnormal detects the disease late and sometimes only by accident. No cost-effectiveness analysis exists. Should anti-Ro52 positivity in PBC prompt routine rheumatological referral? The Danish data suggest a meaningful yield of undiagnosed SjD [23], but generalizability across populations with differing anti-Ro52 prevalence is untested. Does SjD-associated non-erosive polyarthritis ever evolve into erosive RA, or are these biologically distinct trajectories? RF isotype data hint at distinctness [12], but no prospective cohort has followed patients long enough to answer this, and the clinical meaning of subclinical erosion detected by sensitive imaging remains equally unresolved [14]. Underlying all of these is a question about the criteria themselves: whether their application in RF, or antinuclear antibody-positive patients with sicca symptoms, should be formally qualified, so that the exclusion clause acquires operational content rather than being left to a clinical judgment that, in practice, is often not exercised.

Limitations

A single case supports no causal inference and no estimate of generalizability. The temporal association between reclassification, withdrawal of tofacitinib and methotrexate, introduction of ursodeoxycholic acid and hydroxychloroquine, and subsequent remission is consistent with our interpretation but does not establish it; spontaneous improvement cannot be excluded. The initial RA evaluation was reconstructed from external records: swollen and tender joint counts, composite disease activity scores and musculoskeletal imaging beyond plain radiography were never performed, so we cannot state with certainty whether definite clinical synovitis was present at the original assessment. That absence is itself part of what the case documents, but it limits how firmly the original diagnosis can be adjudicated. A formal roussel eclaf causality assessment method (RUCAM) was not applied to the hepatic event, and no HLA typing was performed. The review component is narrative rather than systematic: it was not registered, the search was confined to a single database and to English-language publications, screening was not performed in duplicate, and no risk-of-bias assessment or quantitative synthesis was undertaken. It should be read as an orientation to the field rather than a comprehensive appraisal of it.

## Conclusions

A patient carried a diagnosis of rheumatoid arthritis for three years and was treated with two disease-modifying agents before diagnostic reassessment established Sjögren’s disease with associated non-erosive inflammatory polyarthralgia and coexisting primary biliary cholangitis. Retrospective review suggested that the initial diagnosis had relied primarily on the score-based components of the 2010 ACR/EULAR classification criteria. However, documentation of the required prerequisite of clinical synovitis, as well as exclusion of a more appropriate alternative diagnosis, could not be confirmed from the available medical records, and sicca symptoms that would have prompted further investigation had not been elicited.

In patients with ACPA-negative, RF-positive polyarthritis without radiographic erosions, a structured inquiry into sicca symptoms should be considered. Anti-Ro/SSA testing and, in the presence of abnormal cholestatic liver enzymes, anti-mitochondrial antibody testing may facilitate recognition of Sjögren’s disease and coexisting primary biliary cholangitis before escalation to biologic or targeted synthetic therapy. Early recognition of this overlap may allow appropriate disease-specific treatment with ursodeoxycholic acid and hydroxychloroquine while avoiding unnecessary immunosuppression. Classification criteria are designed to assemble comparable study populations; they support classification but should not replace clinical diagnosis.
